# Exploring psychological safety in healthcare teams to inform the development of interventions: combining observational, survey and interview data

**DOI:** 10.1186/s12913-020-05646-z

**Published:** 2020-08-31

**Authors:** Róisín O’Donovan, Eilish McAuliffe

**Affiliations:** Centre for Interdisciplinary Research, Education, and Innovation in Health Systems (IRIS), School of Nursing, Midwifery & Health Systems, Health Sciences Centre, University College Dublin, Dublin 4, Ireland

**Keywords:** Psychological safety, Mixed methods, Healthcare teams

## Abstract

**Background:**

Psychological safety allows healthcare professionals to take the interpersonal risks needed to engage in effective teamwork and to maintain patient safety. In order to improve psychological safety in healthcare teams, an in-depth understanding of the complex and nuanced nature of psychological safety is needed. Psychological safety concepts, including voice, silence, learning behaviour, support and familiarity, informed the current study’s investigation of psychological safety. This study aims to use a mixed-methods approach to develop an in-depth understanding of psychological safety within healthcare teams and to build on this understanding to inform the development of future interventions to improve it.

**Methods:**

Survey, observational and interview data are triangulated in order to develop an in- depth understanding of psychological safety within four healthcare teams, working within one case study hospital. The teams taking part included one multidisciplinary and three unidisciplinary teams. Observational and survey data were collected during and immediately following team meetings. Individual interviews were conducted with 31 individuals across the four teams. Thematic analysis was used to analyse these interviews.

**Results:**

Survey results indicated a high level of psychological safety. However, observations and interviews captured examples of silence and situations where participants felt less psychologically safe. Findings from across all three data sources are discussed in relation to voice and silence, learning, familiarity and support.

**Conclusion:**

The results of this study provide a detailed description and in-depth understanding of psychological safety within four healthcare teams. Based on this, recommendations are made for future research and the development of interventions to improve psychological safety.

## Background

Psychological safety is a multi-dimensional, dynamic phenomenon that concerns team members’ perception of whether it is safe to take interpersonal risks at work [1]. It is particularly important within healthcare teams who need to work interdependently to co-ordinate safe patient care within a highly complex, dynamic and high stakes work environment [2]. However, a culture of fear and low psychological safety still exists within healthcare organisations [3–7]. There is a need to develop and implement interventions to improve psychological safety within these teams [8]. The ongoing Covid-19 pandemic has highlighted the importance of psychological safety within healthcare teams. Cultivating psychological safety is necessary in order to enable healthcare teams to collectively redesign processes and services to cope with new challenges, learn from mistakes and implement changes accordingly [9]. In order to improve psychological safety, we must first understand the complexity and nuance of psychological safety within healthcare teams [8, 10].

To date, there has been a paucity of cross-level and multilevel research on psychological safety [10]. This has limited our understanding of psychological safety, the concepts related to it and whether it varies across teams within the same organisation [10]. Within organisational research, collecting different kinds of data on the same phenomenon and triangulating this data can help researchers assess complex phenomena, such as psychological safety, more accurately [8, 11–13]. In the current study we use survey, observational and interview data to develop an in-depth understanding of psychological safety within healthcare teams. Building on this understanding, we aim to inform the development of an intervention to improve psychological safety.

The constructs which informed our study design our outlined below. These include constructs which have been linked to either low or high psychological safety (voice and learning behaviour) and which have been found to support psychological safety (positive interpersonal relationships). These constructs play a particularly important role within the context of healthcare teams.

Psychological safety promotes voice and learning behaviour. Speaking up and voice behaviour are interpersonally risky behaviours which play an important role in healthcare teams [8]. Feeling psychologically safe can enable team members to engage in speaking up behaviour, such as asking questions, pointing out a mistake or near miss and making suggestions for improvement [2, 13–16]. Psychological safety also enables learning behaviours, such as seeking help or feedback [1, 17, 18]. Learning behaviours are integral to healthcare teams’ ability to manage demanding conditions, with rapidly evolving knowledge and practice as well as their ability to learn from failure [17, 18]. When healthcare professionals prioritise patient safety by engaging in speaking up and learning behaviours, it is indicative of their levels of psychological safety [19].

On the other hand, lack of psychological safety inhibits team members from speaking up and causes them to opt for avoidance behaviours, such as silence [20]. It is important to acknowledge that although employees may frequently engage in voice behaviour, they could also be withholding other ideas, suggestions or concerns [20]. This highlights the need to go beyond observable behaviours to explore the nuance and complexities of individuals’ experience of psychological safety. In order to do so, this study uses a combination of survey, observation and interview data to gain a full understanding of psychological safety.

Positive interpersonal relationships drive psychological safety [21, 22]. Within healthcare teams, having positive relationships, effective role models and better teamwork climates encourages healthcare professionals to speak up for safety [19]. Team members relationship with the team leader has been found to influence their sense of psychological safety. For example, when leaders engage in supportive behaviour, such as inclusiveness and openness, they foster psychological safety for other team members [23–25]. Peer support and trust among team members also improves psychological safety within teams [10]. Psychological safety can build across time, increasing as team members become more familiar with one another and have positive experiences of engaging in interpersonally risky behaviour [10, 13, 26, 27]. While positive, supportive and trusting interpersonal relationships can foster psychological safety, it is important to note that psychological safety does not imply a team without any conflict or problems [5]. Psychological safety is needed in order for productive conflict, such as task conflict, to occur [28]. Task conflict concerns disagreements related to differences in viewpoints, ideas and opinions about the task being performed and can result in learning and improved performance [13, 28, 29].

In this study, focusing on a single hospital as a case study, we use a mixed methods approach to further our understanding of psychological safety within four healthcare teams. We combine data collected through surveys, observations and individual interviews to gain a holistic understanding of psychological safety in these teams. Building on this understanding, we aim to inform the development of future interventions to improve psychological safety in healthcare teams.

## Methods

### Research setting and participants

This study was undertaken by the authors as part of a wider body of research aiming to develop an intervention to improve psychological safety in healthcare teams. This research was conducted with healthcare professionals working on one of four healthcare teams from within the same acute, suburban hospital. Three of the teams were unidisciplinary – physiotherapists, nurses and speech and language therapists – and one was multidisciplinary. The authors collaborated closely with hospital management in order to recruit healthcare teams from within the hospital. These teams were selected in collaboration with hospital management using purposive sampling in order to identify different team types as well as teams that held meetings amenable to observation. The lead researcher contacted the leader of each team to ask if their team would be interested in taking part in the team observation and survey. Before beginning observations, team members from within each team were asked to consent to the meeting being observed and to completing the survey following the meeting. For interviews, a combination of purposive sampling and snowball sampling were employed to recruit team members from within each team. After the observation and surveys were complete, the researcher informed the team that anyone who was willing to take part in an interview could contact them (the researcher) or their team leader who would then refer them to the lead researcher. Team members were recruited from across all staff grades and included team leaders as well as senior and junior team members [30]. The total number of participants who took part in each phase of data collection can be found in Table 1.

**Table 1:** Number of participants taking part in each phase of data collection

Category	Observations	Survey	Interviews
Team size	Team A: *n* = 11 Participation Rate: 93% Team B: *n* = 6 Participation Rate: 86% Team C *n* = 14 Participation Rate: 100% Team D: *n* = 7 Participation Rate: 100%	Team A: *n* = 11 Participation Rate: 93% Team B: *n* = 6 Participation Rate: 86% Team C: *n* = 13 Participation Rate: 93% Team D: *n* = 6 Participation Rate: 86%	Team A: *n* = 13 Participation Rate: 100% Team B: *n* = 7 Participation Rate: 100% Team C: *n* = 4 Participation Rate: 28% Team D: *n* = 7 Participation Rate: 100%
Total	38	36	31

The team as a whole were observed during regularly scheduled team meetings. Of those who attended the meeting, there was 100% participation rate in the survey within teams A and B. There was one team members in team C and another in team D who was part of the observations but did not complete a survey. Since surveys were kept completely anonymous, it was not possible to assess whether the same participants who completed the survey also took part in an interview. In teams A (*n* = 2), B (*n* = 1) and D (*n* = 1), participants who were not present during observations and who did not complete a survey were recruited by the team leader to take part in an interview.

### Data collection

Within case study designs, the use of multiple sources of evidence in recommended in order to capture a holistic understanding of the phenomena being studied [31]. The current study triangulates multiple sources of data in order to gain an in-depth understanding of psychological safety in healthcare teams. Rather than adopting the commonly used approach to triangulation to gain more credible or valid results, we draw on a post-modern paradigm which views reality as having multiple, fractured dimensions and being socially constructed. This approach requires acknowledgment that all research findings are shaped by the approach used to collect them and that different methods of data collection will offer different results [32]. Therefore, we use multiple methods to deepen our understanding of psychological safety by encouraging re-interpretation of findings as data sources reveal new insights and, thus, facilitating a more complex and in-depth exploration of healthcare professionals experiences of psychological safety. This approach to triangulation of data has been termed crystallization [33].

#### Composite measure of psychological safety

Survey and observational data were collected using the composite measure developed and presented in a recent paper focused on measuring psychological safety in healthcare teams [34]. For each team, one weekly meeting was observed using the observation measure, which captured voice, silence, supportive or unsupportive and familiarity behaviours. The observational data were collected as part of a pilot test of the measure and, as a result, behaviours were amended following each observation, however the overall categories remained the same. Observations were completed by one researcher who sat at the table or in the room where each meeting was conducted. The observer tracked the behaviours displayed by the team leader and team members by making a mark in the “behaviour count” box for the relevant behaviour. Observations were made by only one researcher in order to reduce the inhibiting effect the presence of two researchers might have on the team’s behaviour. The observed team meetings varied in length. The meeting held by Team A lasted 30 min, Team B’s meeting lasted 90 min, Team C’s meeting lasted 70 min and Team D had a meeting which lasted 120 min. All meeting were the teams regularly scheduled team meetings. Each discussed clinical and/or management issues that were relevant to their team.

Following the meeting, team members were asked to complete the survey component of the composite measure. There were three sections in the survey which assessed participants’ psychological safety in relation to: the team leader, other team members and the team as a whole. As part of a pilot test of the survey, a 7 point Likert scale was used for teams A-C. For team D, a 10 point Likert scale was tested. This was done in order to check if using a 10 point Likert scale would give participants an even wider spectrum of response options and, thus, allow the survey to capture more variability in participants’ responses [35]. Surveys did not ask for any identifiable information and were kept completely anonymous. Participants completed their survey in the same room as one another. There was plenty of space for them to move freely in order to complete their survey in private and surveys were handed directly to the researcher once completed.

#### Interviews

Semi-structured interviews were conducted with 31 participants from across the four teams. The full interview schedule is presented as a supplementary file. This interview data was collected in order to gain an in-depth understanding of individuals’ experience of psychological safety and explore whether there were any emerging differences compared to the team level observations or survey responses. Interviews were conducted in a private room located within the case study hospital and lasted an average of 28 min. A full description of the process used to collect and analyse the interview data can be found in O'Donovan, De Brún & McAuliffe (in preparation). Hybrid inductive-deductive thematic analysis was used to identify themes which correspond to the concepts covered in the observations and survey data. Descriptive, open codes were assigned to each interview. These codes were then reviewed and refined, with reference to the psychological safety literature, in order to identify overarching themes. Analysis also compared findings from individuals in the same team to explore the consistencies and inconsistencies across cases. Thematic analysis was chosen because it is a theoretically flexible approach to qualitative analysis which allows the combination of inductive and deductive methods [36, 37]. As highlighted by Braun and Clarke [36], thematic analysis is a useful method for working within a participatory research paradigm, with participants as collaborators, and for producing qualitative analysis which can inform policy development. Since the overarching aim of this study was to inform the development of an intervention to improve psychological safety which is grounded in the experiences of healthcare professionals, thematic analysis was particularly suitable. Our analysis focused on themes which captured participants’ experiences of speaking up or remaining silent, engaging in learning behaviour and their experience of support, or lack of support and familiarity within the team.

### Ethics

Ethical approval was obtained for this study from the Human Research Ethics Committee in University College Dublin (Reference number: LS-17-67). Written informed consent was obtained from all participants prior to each stage of data collection. In order to maintain anonymity, no identifiable information was collected during observations or surveys. Interviews were assigned a code made up of P (participant), interview number (e.g. the first interview conducted within each team was given the number 1) and team letter (A, B, C or D) and any identifiable characteristics were removed from the interview transcripts.

## Results

### Team A

#### Survey results

All survey responses are displayed in Table 2. They indicated that team members felt psychologically safe. In team A, a mean response of 6.700 was given for section 1, 6.597 for section 2 and 6.212 for section 3.

**Table 2:** Survey Results

Section 1 Psychological safety related to team leader				
Questions	Team A response	Team B response	Team C response	Team D response^b^
1. If I had a question or was unsure of something in relation to my role at work, I could ask my team leader	7: 90.09% (*n* = 10) Missing: 9.1% (*n* = 1)^a^	7: 100% (*n* = 6)	6: 46.2% (*n* = 6) 7: 46.2% (*n* = 6) Missing: 7.7% (*n* = 1)^a^	7: 16.7% (*n* = 1) 8: 16.7% (*n* = 1) 10: 33.3% (*n* = 2) Missing: 33.3% (*n* = 2)^a^
2. I can communicate my opinions about work issues with my team leader	7: 81.8% (*n* = 9) 6: 9.1%(*n* = 1) Missing: 9.1% (*n* = 1)^a^	7: 100% (*n* = 6)	6: 38.5% (*n* = 5) 7: 53.8% (*n* = 7) Missing: 7.7% (*n* = 1)^a^	6: 16.7% (*n* = 1) 7: 16.7% (*n* = 1) 8: 16.7% (*n* = 1) 10: 16.7% (*n* = 1) Missing: 33.3% (*n* = 2)^a^
3. I can speak up about personal problems or disagreements to my team leader	6: 27.3% (*n* = 3) 7: 63.3% (*n* = 7) Missing: 9.1% (*n* = 1)^a^	5: 16.7%(*n* = 1) 6: 16.7%(*n* = 1) 7: 66.7%(*n* = 4)	5: 7.7% (*n* = 1) 6: 30.8% (*n* = 4) 7: 53.8% (*n* = 7) Missing: 7.7% (*n* = 1)^a^	4: 16.7% (*n* = 1) 8: 16.7% (*n* = 1) 9: 16.7% (*n* = 1) 10: 16.7% (*n* = 1) Missing: 33.3% (*n* = 2)^a^
4. I can speak up with recommendations/ideas for new projects or changes in procedures to my team leader	6: 27.3% (*n* = 3) 7: 63.6% (*n* = 7) Missing: 9.1% (*n* = 1)^a^	6: 16.7% (*n* = 1) 7: 83.3% (*n* = 5)	6: 46.2% (*n* = 6) 7: 46.2 (*n* = 6) Missing: 7.7% (*n* = 1)^a^	6: 16.7% (*n* = 1) 7: 16.7% (*n* = 1) 10: 33.3% (*n* = 2) Missing: 33.3% (*n* = 2)^a^
5. If I made a mistake on this team, I would feel safe speaking up to my team leader	5: 9.1% (*n* = 1) 6: 9.1% (*n* = 1) 7: 72.7% (*n* = 8) Missing: 9.1% (*n* = 1)^a^	7: 83.3% (*n* = 5) Missing: 16.7% (*n* = 1)^a^	6: 46.2% (*n* = 6) 7: 46.2% (*n* = 6) Missing: 7.7% (*n* = 1)^a^	6: 16.7% (*n* = 1) 10: 50.0% (*n* = 3) Missing: 33.3% (*n* = 2)^a^
6. If I saw a colleague making a mistake, I would feel safe speaking up to my team leader	5: 18.2%(*n* = 2) 6: 18.2% (*n* = 2) 7: 54.5% (*n* = 6) Missing: 9.1% (*n* = 1)^a^	6: 16.7% (*n* = 1) 7: 83.3% (*n* = 5)	6: 30.8% (*n* = 4) 7: 53.8% (*n* = 7) Missing: 15.4% (*n* = 2)^a^	6: 33.3% (*n* = 2) 8: 16.7% (*n* = 1) 10: 16.7% (*n* = 1) Missing: 33.3% (*n* = 2)^a^
7. If I speak up/voice my opinion, I know that my input is valued by my team leader	5: 9.1% (*n* = 1) 6: 9.1% (*n* = 1) 7: 72.7% (*n* = 8) Missing: 9.1% (*n* = 1)^a^	6: 16.7% (*n* = 1) 7: 83.3% (*n* = 5)	5: 7.7% (*n* = 1) 6: 61.5% (*n* = 8) 7: 23.1% (*n* = 3) Missing: 7.7% (*n* = 1)^a^	6: 16.7% (*n* = 1) 7: 16.7% (*n* = 1) 10: 16.7% (*n* = 1) Missing: 50.0% (*n* = 3)
8. My team leader encourages and supports me to take on new tasks or to learn how to do things I have never done before.	6: 18.2% (*n* = 2) 7: 72.7% (*n* = 8) Missing: 9.1% (*n* = 1)^a^	7: 100% (*n* = 6)	6: 38.5% (*n* = 5) 7: 53.8% (*n* = 7) Missing: 7.7% (*n* = 1)^a^	5: 16.7% (*n* = 1) 6: 16.7% (*n* = 1) 10: 33.3% (*n* = 2) Missing: 33.3% (*n* = 2)^a^
9. If I had a problem in this company, I could depend on my team leader to be my advocate	7: 90.9% (*n* = 2) Missing: 9.1% (*n* = 1)^a^	6: 16.7% (*n* = 1) 7: 83.3% (*n* = 5)	6: 38.5% (*n* = 5) 7: 46.2% (*n* = 6) Missing: 15.4% (*n* = 2)	6: 16.7% (*n* = 1) 8: 16.7% (*n* = 1) 10: 33.3% (*n* = 2) Missing: 33.3% (*n* = 2)
Section 2 Please answer the following questions in relation to your peers/the other members of your team				
Questions	Team A	Team B	Team C	Team D
10. If I had a question or was unsure of something in relation to my role at work, I could ask my peers	6: 9.1% (*n* = 1) 7: 90.9% (*n* = 10)	6: 33.3 (*n* = 2) 7: 66.7% (*n* = 4)	6: 84.6% (*n* = 11) 7: 15.4% (*n* = 2)	6: 16.7% (*n* = 1) 8: 16.7% (*n* = 1) 10: 66.7% (*n* = 4)
11. I can communicate my opinions about work issues with my peers	6: 9.1% (*n* = 1) 7: 90.9% (*n* = 10)	6: 16.7% (*n* = 1) 7: 83.3% (*n* = 5)	5: 7.7% (*n* = 1) 6: 61.5% (*n* = 8) 7: 30.8% (*n* = 4)	6: 16.7% (*n* = 1) 8: 50.0% (*n* = 3) 9: 16.7% (*n* = 1) 10: 16.7% (*n* = 1)
12. I can speak up about personal issues to my peers	5: 9.1% (*n* = 1) 6: 36.4% (*n* = 4) 7: 54.5% (*n* = 6)	4: 16.7% (*n* = 1) 5: 16.7% (*n* = 1) 6: 16.7% (*n* = 1) 7: 50.0% (*n* = 3)	4: 7.7% (*n* = 1) 5: 38.5% (*n* = 5) 6: 30.8% (*n* = 4) 7: 23.1% (*n* = 3)	5: 16.7% (*n* = 1) 7: 33.3% (*n* = 2) 8: 50.0% (*n* = 3)
13. I can speak up with recommendations/ideas for new projects or changes in procedures to my peers	6: 45.5% (*n* = 5) 7: 54.5% (*n* = 6)	6: 16.6% (*n* = 1) 7: 83.3% (*n* = 5)	5: 7.7% (*n* = 1) 6: 53.8% (*n* = 7) 7: 38.5% (*n* = 5)	6: 16.7% (*n* = 1) 7: 16.7% (*n* = 1) 8: 16.7% (*n* = 1) 9: 16.7% (*n* = 1) 10: 33.3% (*n* = 2)
14. If I made a mistake on this team, I would feel safe speaking up to my peers	5: 9.1% (*n* = 1) 6: 36.4% (*n* = 4) 7: 54.4% (*n* = 6)	5: 16.7% (*n* = 1) 6: 50.0% (*n* = 3) 7: 33.3% (*n* = 2)	4: 7.7% (*n* = 1) 5: 23.1% (*n* = 3) 6: 46.2% (*n* = 6) 7: 23.1% (*n* = 3)	6: 16.7% (*n* = 1) 7: 33.3% (*n* = 2) 8: 33.3% (*n* = 2) 10: 16.7% (*n* = 1)
15. If I saw a colleague making a mistake, I would feel safe speaking up to this colleague	5: 9.1% (*n* = 1) 6: 45.5% (*n* = 5) 7: 45.5% (*n* = 5)	5: 16.7% (*n* = 1) 6: 50.0% (*n* = 3) 7: 33.3% (*n* = 2)	4: 15.4% (*n* = 2) 5: 7.7% (*n* = 1) 6: 61.5% (*n* = 8) 7: 15.4% (*n* = 2)	6: 33.3% (*n* = 2) 7: 16.7% (*n* = 1) 8: 16.7% (*n* = 1) 10: 33.3% (*n* = 2)
16. If I speak up/voice my opinion, I know that my input is valued by my peers	5: 9.1% (*n* = 1) 6: 27.3% (*n* = 3) 7: 63.6% (*n* = 7)	5: 16.7% (*n* = 1) 6: 50.0% (*n* = 3) 7: 33.3% (*n* = 2)	4: 7.7% (*n* = 1) 5: 69.2% (*n* = 9) 7: 23.1% (*n* = 3)	6: 16.7% (*n* = 1) 7: 16.7% (*n* = 1) 9: 50.0% (*n* = 3) 10: 16.7% (*n* = 1)
Section 3 Please answer in relation to your team as a whole				
Questions	Team A	Team B	Team C	Team D
17. It is difficult to ask other members of this team for help	1: 9.1% (*n* = 1) 5: 9.1% (*n* = 1) 7: 63.6% (*n* = 7)	1: 33.3% (*n* = 3) 6: 33.3% (*n* = 2) 7: 33.3 (*n* = 2)	2: 23.1% (*n* = 3) 3: 7.7% (*n* = 1) 4: 7.7% (*n* = 1) 5: 15.4% (*n* = 2) 6: 30.8% (*n* = 4) 7: 15.4% (*n* = 2)	2: 16.7% (*n* = 1) 7: 33.3% (*n* = 2) 10: 50.0% (*n* = 3)
18. People keep each other informed about work-related issues in the team	5: 27.3% (*n* = 3) 6: 27.3% (*n* = 3) 7: 45.5% (*n* = 5)	3: 16.7% (*n* = 1) 6: 50.0% (*n* = 3) 7: 33.3% (*n* = 2)	3: 15.4% (*n* = 2) 5: 15.4% (*n* = 2) 6: 69.2% (*n* = 9)	3: 16.7% (*n* = 1) 4: 16.7% (*n* = 1) 8: 33.3% (*n* = 2) 9: 16.7% (*n* = 1) 10: 16.7% (*n* = 1)
19. There are real attempts to share information throughout the team	5: 9.1% (*n* = 1) 6: 36.4% (*n* = 4) 7: 54.5% (*n* = 6)	6: 50.0% (*n* = 3) 7: 50.0% (*n* = 3)	3: 7.7% (*n* = 1) 5: 15.4% (*n* = 2) 6: 53.8% (*n* = 7) 7: 23.1% (*n* = 3)	3: 16.7% (*n* = 1) 4: 16.7% (*n* = 1) 8: 16.7% (*n* = 1) 9: 16.7% (*n* = 1) 10: 33.3% (*n* = 2)

^a^The team leader did not answer section 1 as the questions were not applicable to her role

^b^The survey administered for team D had been updated based on pilot test results and used a 10 point likert scale. For full details see O’Donovan et al. [34]

#### Observations

A positive, constructive atmosphere was observed during the team meeting. While the team leader spoke the most, team members were given opportunities to speak up. However, five to six team members dominated the discussion and not all team members spoke. These observations indicated that both team members and team leaders engaged in voice, learning, supportive and familiarity behaviours. There were no counts of defensive voice, silence or unsupportive behaviour. The specific behaviours displayed can be seen in Table 3 and observer ratings can be seen in Table 4.

**Table 3:** Team Observations Results

Behaviour	Team A Behaviour Count		Team B Behaviour Count		Team C Behaviour Count		Team D Behaviour Count	
	Team Member	Team Leader	Team Member	Team Leader	Team Member	Team Leader	Team Member	Team Leader
**Voice**	**14**	**12**	**30**	**19**	**22**	**20**	**33**	**25**
Communicating opinions to others even if they disagree	1	0	2	0	2	0	3	2
Asking open questions	4	6	8	7	8	5	18	4
Providing Information	NA	NA	NA	NA	NA	NA	10	12
Providing feedback	7	4	10	10	8	10	0	2
Providing help or solutions	2	2	10	2	4	5	0	5
Correcting others	NA	NA	NA	NA	NA	NA	2	0
**Defensive Voice**	**0**	**0**	**1**	**0**	**0**	**0**	**0**	**0**
Denying faults or blame others	0	0	1	0	0	0	0	0
Showing aggression (Raising voice, large gestures)	0	0	0	0	0	0	0	0
Evading confrontation by focusing only on positives	0	0	0	0	0	0	0	0
**Silence**	**0**	**0**	**2**	**0**	**0**	**0**	**1**	**2**
Facial expression indicates fear	0	0	0	0	0	0	0	0
Facial expression indicates disengagement	0	0	1	0	0	0	1	2
Closed body language (arms closed, lean backwards)	0	0	1	0	0	0	0	0
No eye contact (with speaker)	0	0	0	0	0	0	NA	NA
**Supportive**	**12**	**14**	**13**	**43**	**23**	**13**	**29**	**21**
Sharing procedures, knowledge and experience	4	9	4	7	11	6	14	3
Active listening (verify, paraphrase)	3	0	3	7	6	0	1	0
Acknowledging achievements/ congratulating one another	NA	NA	2	2	1	2	0	2
Use of inclusive language such as “we”	0	3	0	20	3	5	4	3
Agreeing/Responding positively or enthusiastically to input	5	2	4	7	3	2	2	3
Leaders words and deeds align	NA	0	NA	0	NA	0	NA	NA
Sharing future plans	NA	NA	NA	NA	0	4	8	10
Delegating tasks	NA	NA	NA	NA	NA	NA	1	4
**Unsupportive**	**0**	**0**	**5**	**1**	**1**	**0**	**0**	**1**
Interrupting	0	0	2	0	0	0	0	1
Discussions within small sub-groups	0	0	1	0	1	0	0	0
Reacting cold/ignoring a joke	0	0	2	1	0	0	0	0
**Learning or Improvement**	**8**	**12**	**16**	**15**	**9**	**10**	**16**	**17**
Reviewing own progress and performance	1	0	2	3	1	1	1	3
Asking for feedback	3	1	1	1	0	0	0	3
Asking for help or solutions	1	1	0	1	0	0	2	0
Asking for input from all meeting participants	0	3	1	5	0	5	2	4
Informing the team about issues or mistakes related to patient safety	2	6	12	2	4	3	5	4
Looking for improvement opportunities and speaking up with ideas	1	1	0	3	4	1	5	3
Acknowledging own mistakes	NA	NA	NA	NA	NA	NA	1	0
**Familiarity**	**10**	**4**	**9**	**6**	**8**	**4**	**10**	**8**
Talking about personal, non-work matters (with team members)	2	1	0	0	0	0	1	1
Talking about personal, non-work matters (with team leader)	2	0	0	0	0	0	0	0
Laughing about a joke	6	3	9	6	8	4	9	7

**Table 4:** Observer Ratings

Observations	Team A	Team B	Team C	Team D
There was enough opportunity for participants to ask for help	Strongly agree	Strongly agree	Agree	Strongly agree
There was enough opportunity for participants to speak up	Strongly agree	Strongly agree	Somewhat agree	Strongly agree
There was enough opportunity for participants to discuss with the team leader	Strongly agree	Strongly agree	Agree	Strongly agree
Certain team members dominated the discussion	Somewhat disagree	Disagree	Agree	Disagree
Decisions were made together, by the entire team	Agree	Somewhat agree	Somewhat disagree	Agree
The atmosphere in this team was constructive	Strongly agree	Agree	Agree	Agree
People seemed genuine and not to hold back anything	Strongly agree	Somewhat disagree	Neither agree or disagree	Agree

### Interviews

#### Voice and silence

Team members described an open team atmosphere where they felt listened to, respected and psychologically safe. They felt comfortable speaking up about work issues or things *“they felt very strongly”* about and would go to their team leader if they were *“frustrated”* or *“struggling”*. Interviews highlighted that there were opportunities to speak up during meetings. However, team members remained silent about certain issues. Some team members believed that discussing conflict, personal or confidential issues within a team setting may not be *“appropriate”*. They would discuss these issues outside the team setting instead.

*“but I would say maybe it’s when the group disassembles that some of those opinions come out, you know, it mightn’t always be that effective.”*

Conflict was *“pushed under the carpet”* because team members wanted to avoid insulting or questioning others or didn’t want to *“rock the boat or cause issues”*. Conflict avoidance was linked to small team size which meant that *“everybody knows each other”* and it would *“make it harder for yourself”* to speak up about conflict. One junior team member felt uncomfortable speaking up about confrontational issues with more experienced team members due to a fear that they would be dismissive and think *“sure what does {team member} know”*. However, junior team members all felt comfortable or *“confident”* asking for help.

A senior member of team A suggested that explicitly asking for input from junior team members could improve psychological safety and speaking up.

*“looking for people’s opinions rather than waiting for somebody to offer, like asking, maybe some of the younger members, because I actually do think their opinion is really valuable.”*

#### Learning

A relaxed atmosphere was deliberately cultivated to encourage learning behaviour.

*“we’re very aware of trying to create an environment, em, like relaxed environment because you know when they’re relaxed, they’re going to learn more, they’ll ask more questions”*

Team members considered patient care to be their *“focus”* and felt they could speak up about patient safety issues. They recognised that speaking up about errors was important for learning and improvement within the team.

*“there’s lots of different failures in the system that probably will lead to that happening again but it is important that we try, I suppose, to remedy them.”*

Members of team A talked about their team having a solution-focused approach to problem solving and highlighted that the team’s supervision model created a psychologically safe space for learning. The team leader advocated for the importance of trying new things and learning from them. However, three team members said there was not enough time for learning and there was a need to schedule time for supervision, where concerns and ideas could be voiced.

*“it’s just something that kind of does get put on the longer finger a little bit, so that, it probably would be good to actually have time like an actual scheduled time to do that.”*

#### Support

Members of team A talked about feeling supported by their team leader and their peers. This encouraged them to speak up.

*“you can go to the group and they’ll have your back, in terms of, yeah, your kind of professional role.”*

The team leader provided support in relation to career development as well as personal and work needs. Team members were confident they would be supported by their leader if/when they asked for it.

*“whatever small little rubbish is going on in your life, she will take that, you know, really into consideration and she is really so much about the staff member.”*

#### Familiarity

Familiarity between team members facilitated psychological safety. Team members found it easier to speak openly as they got to know one another better and worked together for longer.

*“Yeah she’s very easy to talk to… because I’ve worked with her for about X years.”.*

Lack of familiarity had a negative impact on team members’ feelings of psychological safety. One team member identified themselves as being new to the team. In addition, team members whose roles were more separate from the rest of the team felt less comfortable.

*“I probably wouldn’t feel as comfortable getting involved in some of their discussions.”*

Team members highlighted the need to build relationships with those who work in these separate roles.

### Team B

#### Survey

Survey results indicated that team members felt psychologically safe. Participants gave a mean response of 6.750 for section 1, 6.405 for section 2 and 5.667 for section 3.

#### Observations

At first, the team leader gave feedback, then each team member had an opportunity to contribute. At the end of the meeting, team members were given an opportunity to raise any concerns. There were some tense moments where there may have been an undertone of confrontation. Jokes were used to defuse these moments. During these instances the observer felt that people could be holding back. All participants engaged in voice, supportive, learning and familiarity behaviours but also displayed unsupportive behaviours. Team members displayed defensive voice and silence behaviours.

### Interviews

#### Voice and silence

According to interviews, the leader of team B created an open, inclusive team atmosphere which made team members feel psychologically safe.

*“it’s an open forum and I’ve never really felt that I couldn’t say anything.”*

Team members prioritised patients and would speak up about patient safety issues.

*“because I would kind of be out for the patient, you know, so I would have enough {confidence}, to say that’s not acceptable, or it’s not acceptable behaviour.”*

However, according to the team leader, meetings could be *“more participative”* without certain team members. This suggests that the presence of these team members reduces psychological safety for others. Participants described negative reactions to people speaking up during meetings, such as *“tut tutting”*, *“rolling eyes”* or *“sighing”*. The team leader highlighted the need for improving people’s behaviour during team meetings.

*“I suppose people are less likely to contribute if they feel like that’s a risky response or there is the risk of that being a response.”*

According to the team leader, improving psychological safety would involve making it clear to all team members that they play a valuable role in the team.

*“it’s to convince, like everybody at that table has a critical role to play.”*

Similar to Team A, conflict, personal or confidential issues were not deemed *“appropriate”* for the group setting and were discussed outside of team meetings. While conflict existed within the team it was not *“open”* and was *“sometimes ignored”*. Team members would withhold their *“true feelings”* to try to *“keep the peace”*, *“incubate the mess in front of everybody”* and to avoid making other team members feel attacked. When asked why conflict isn’t addressed, the team leader said, *“we’re not there yet”*.

#### Learning

Team members felt comfortable admitting mistakes and considered it to be the *“whole point of the forum {team meeting}”* and necessary for learning.

*“you can’t fix them if you don’t highlight them.”*

However, they also referred to individuals who have not admitted when they haven’t done something and have covered it up because *“they don’t like to show up they’re not doing {something}.”*

Interviews indicated that the team was going through an *“evolution”* and trying to become more focused on learning. This involved having dedicated time within and outside regular team meeting to discuss errors and concerns. According to the team leader, this improved speaking up and psychological safety in the team.

*“We have had people say ‘I completely messed up’.”*

#### Support

Team members said that their leader is *“100% behind you”* and that leadership support played an important role in creating a psychologically safe environment.

*“I think too it’s down to having the confidence in our leaders, in our leader as well. That you know that it’s kind of a safe space to talk.”*

There was one reference to lack of peer support on the team. According to one participant, another team member has complained about a lack of support within the team, but this individual has not given support to others. This presented peer support as a reciprocal relationship between members.

*“he wants support, he’s not giving support on the other side of it, he’s not giving support to us.”*

One team member said they have received support in the form of other team members’ expertise.

*“I’m not the expert in that field, I would be kind of guided by our {lists specific roles}.”*

#### Familiarity

Most team members have worked in the hospital for long enough to be familiar with their colleagues. This made it easier for them to speak up.

*“maybe because I’m here so long that maybe it’s a thing with age (laughs). You know, I don’t have a problem really in that kind of a setting speaking.”*

There were three team members who identified themselves as being new to the team. A new team member didn’t feel the same level of comfort as others because she felt the team didn’t know her well enough. This team member highlighted the need for time with the team in order for her to become more comfortable.

*“I’m still not 100% comfortable, I don’t think they know me yet.”*

### Team C

#### Survey results

Survey results indicated that team members felt psychologically safe. Participants gave a mean response of 6.611 for section 1, 6.064 for section 2 and 5.308 for section 3.

#### Observations

There was a collaborative, inclusive and constructive atmosphere during the team meeting. While there were opportunities for participants to speak up, certain individuals dominated the discussions. All participants displayed voice, supportive, learning and familiarity behaviours. Team members displayed one count of unsupportive behaviour. There were no defensive voice or silence behaviours recorded.

### Interviews

#### Voice and silence

Interviews referenced a historical culture of fear which lead to a lack of honesty and low psychological safety. One team member said that it has been difficult to change this culture completely, since the same people are still working there. As a result, some of this culture remained and team members reported silence and a lack of encouragement to speaking up.

*“I think overall, the consensus was not to speak, there was never encouragement to speak so I can’t think of any occasion where I actually felt comfortable, there may have been occasions where I became so frustrated, that then I would have, you know, given my opinions, but that would not have been done in a comfortable environment.”*

Position in the hierarchy influenced speaking up behaviour. The team leader was aware that team members may remain silent because of her role as leader. According to one team member, there was a reluctance to speak up when the team leader was present. This team member thought that there would be more open discussions and more things would *“come out”* if they could run their own meetings and meet their leader less regularly.

*“they don’t want to say with the management there, because it will show them to be not coping as well.”*

However, Team C was going through a *“transition”* and was changing from the historically *“negative”* culture. The team leader aimed to make the team more inclusive and let ideas *“come from them {team members} up”*. Team members felt psychologically safe with the leader because she is *“reasonable”* and they felt valued by her.

*“I actually would be 100% confident, that if I did have to challenge any of her, em, any concerns that there wouldn’t be a problem that she’s extremely reasonable and yeah.”*

Team members discussed patient safety and actively raised issues in order to have an open discussion and avoid creating tension or misunderstandings.

*“I can voice it and everyone knows what my opinion is and I know what other people’s opinion is rather than them go to a one to one, and say, I don’t agree with x, y and z, because I think that’s kind of going behind people’s backs, in a certain way.”*

However, like the other teams, they would discuss personal or confidential issues outside of the team setting.

#### Learning behaviour

The team leader said that by speaking up about mistakes, she could foster trust and encourage other team members to do the same.

*“so I think the more they see me owning up to mistakes, the more they’re going to trust me {…} and the more they’ll own up to mistakes.”*

One team member referred to the team as being very *“pro-learning”* and that team members were encouraged to speak up so they could learn from every *“meeting or conflict”*.

*“they’re very much encouraging you to say it, it’s a safe space we’re not going to go back and, and talk about it, and we can learn, because I might be struggling with something, that someone else mentions, and I go, ‘oh god, I’m actually struggling with that area too, how are you going about it?’”*

#### Support

Team members referred to the leader being supportive, inclusive and *“open”*. One team member has learned from experience that she was more likely to get support if she approached team members one to one, rather than in a group setting

*“I realised you’re better off actually going from one to one to one before than bringing it up {in a group setting}, and then you might get some support behind you.”*

#### Familiarity

Familiarity between team members and the fact that they were all part of the same discipline, encouraged psychological safety.

*“most of us have worked together for a while so we know what’s going on.”*

One team member identified themselves as being new to the team. There was a lack of familiarity between the team leader and the other team members and there was still a need for trust to build between them.

*“going to take a while though, {…} for me to be able to say to you, you know, tell you what, they do trust me and we’re open.”*

One team member said that if the team had been given more opportunities to become familiar with one another when they first joined the team, they would feel more comfortable within team meetings.

*“to get more familiar with each other and not to be as worried about somebody else being in the room.”*

### Team D

#### Survey

Survey results indicated that team members felt psychologically safe. Based on pilot testing, survey responses were altered to provide participants with a wider spectrum of response options (O’Donovan et al., in press). Responses could be between 1 and 10, 1 being “strongly disagree” and 10 being “strongly agree”. Participants gave a mean response of 7.704 for section 1, 8.071 for section 2 and 7.333 for section 3.

#### Observations

There was a positive, friendly and constructive atmosphere during the team meeting. While some tension was noted between the senior team members, there were opportunities for speaking up and most team members communicated openly. Decisions were made together, and team members seemed not be holding anything back. All participants displayed voice, silence, supportive, learning and familiarity behaviours. There was one count of unsupportive behaviour for the team leader.

### Interviews

#### Voice and silence

All team members felt there were opportunities to speak up within team meetings and gave examples of speaking up about work related issues, including patient safety issues.

*“the staff meeting, the biggest decisions are made at those and I think everyone gets an opportunity to weigh in.”*

However, team members also noted that meetings mostly focused on operational issues. As a result, the issues that the team members wanted to raise were not given time.

*“the bits that maybe us minions (laughs) want to talk about is back loaded.”*

Participants suggested building time into meetings to discuss team members’ concerns and for them to connect in *“a meaningful way”* and understand where one another’s *“emotions are at on a daily basis”* in order to provide support and reassurance.

There was more pressure when raising issues in the group and so team members would speak to the leader about personal issues or would discuss conflict with one other team member.

*“I would be more likely to bring things to my {team leader}, or to talk to people at an individual level em, if it was something sort of, I don’t know, beyond those boundaries.”*

The majority of team members’ silence occurred when they gathered together as a group. Conflict or disagreements happened *“under the covers”* or got *“brushed away”* and were not discussed openly. Team members said they remained silent in team meetings to be polite and respectful to one another. They position this as a functional way to maintain good working relationships.

*“so yeah, I think politeness really underpins a lot of our interactions.”*

The influence of hierarchy and experience was also noted. Senior members were aware that junior team members were less likely *“to rock the boat or make suggestions”*. While junior team members were more nervous about speaking up, they became more psychologically safe as they gained experience.

*“knowing what’s too much for one person really helped me figure out when to kind of just get on with it or when to kind of ask around.”*

#### Learning

There were team members who remained silent about their ideas for change rather than risk sharing them with the team.

*“don’t know if I’d feel comfortable suggesting too much change, just because I’d feel like that’s not really my remit or you know, everything, there’s a lot of well-established routines and I wouldn’t really be one to rock the boat too much.”*

However, participants commented that the team has improved and become more open to learning and change. One member said it was easier for them to speak up and share their opinions during the meeting which was observed as part of this study.

*“people were speaking up and we were able to push what was going to work best for us across.”*

#### Support

Examples were given of peer support. One participant referred to being supported during a difficult experience, explained that the team is very supportive, and that providing support is part of their jobs.

*“I think this is one of the most supportive teams that I have ever experienced, em like I said there is nobody on the team that I haven’t spoken to about one thing or another.”*

The leader was described as approachable, inclusive and supportive. Receiving support from the team leader made participants *“confident in their choice”* to speak up and share their opinion.

#### Familiarity

Familiarity between team members influenced psychological safety. They were more comfortable speaking to the team members who they worked closely with or considered good friends. There was one team member who identified themselves as being new to the team. One participant said that she would feel more *“conscious”* of what she was saying to team members who she didn’t work closely with, suggesting lower psychological safety. Others said their psychological safety improved as they got to know their colleagues better.

*“I’ve become much more comfortable as I’ve gotten to know people to ask for help.”*

## Discussion

**Fig. 1:**
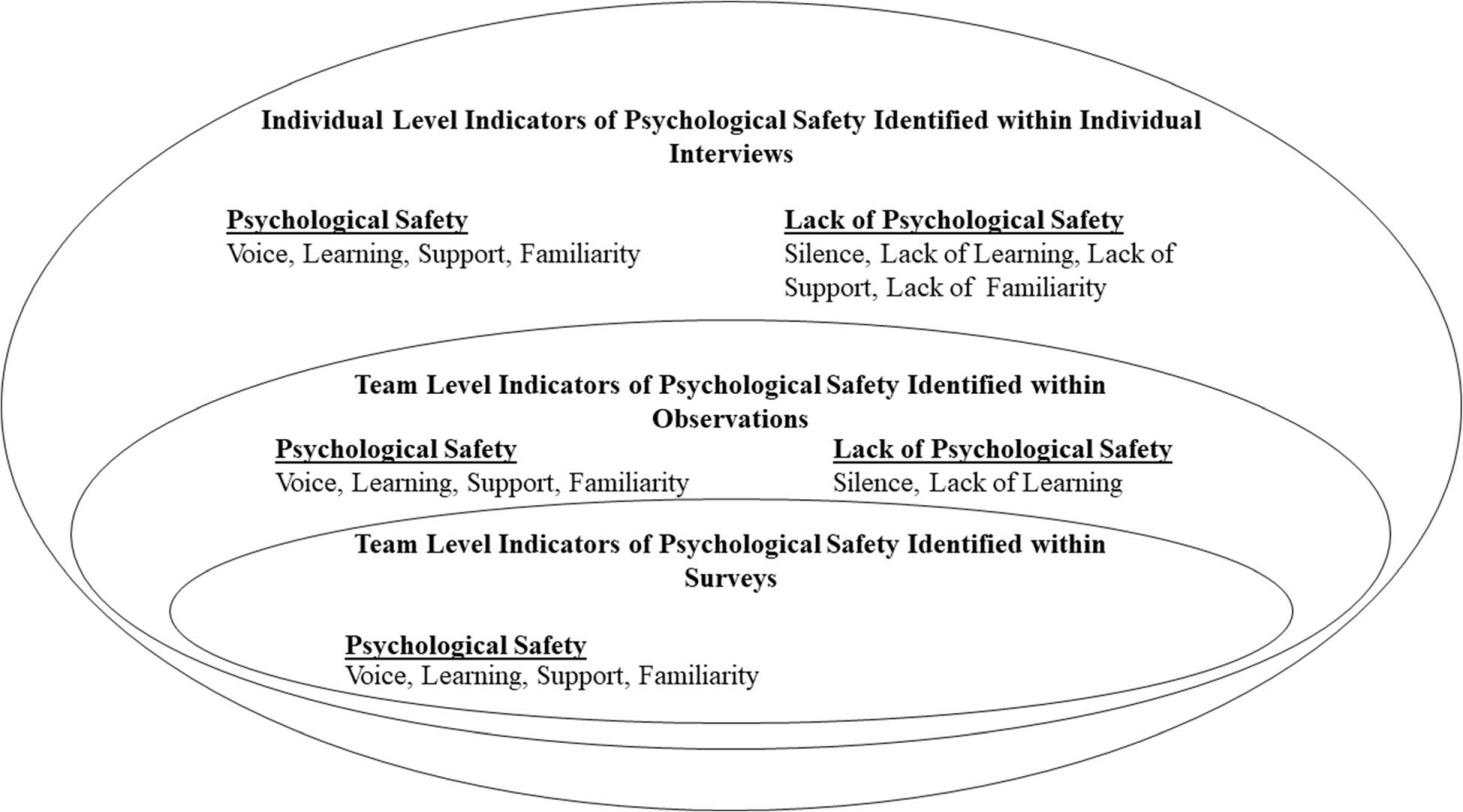
Indicators of Psychological Safety Identified at the Team and Individual Levels. This figure illustrated the key findings coming from each data collection source. Positive and negative indicators of psychological safety are indicated in relation to voice, learning, support and familiarity

This study provides an in-depth understanding of psychological safety within four healthcare teams working in the same case study hospital. It addresses recent calls for the use of multiple methods to capture a more accurate and nuanced understanding of psychological safety in healthcare teams [8, 10]. While survey results from each team reflected high psychological safety, observations and interviews captured exceptional cases and more subtle dynamics within teams. Observations captured behaviours displayed during team meetings while interviews offered an insight into psychological safety both within and outside meetings, as well as within a historical context. An overview of the results can be seen in Fig. 1. In this figure, we aim to highlight the indicators of psychological safety identified at both team and individual levels. Figure 1 also illustrates the added degree of variance in psychological safety captured by observations and interviews, in comparison to survey results. While survey results indicated medium to high levels of psychological safety within all teams, both observations and interviews captured examples of silence and an absence of learning behaviour. In addition, interviews highlighted examples of low levels of support from other team members and a lack of familiarity between certain team members. Survey and observation results captured team level dynamics which gave context to our understanding of individual level perceptions of psychological safety. This was particularly important, given that the overarching aim of this research is to inform the development of a team-level intervention to improve psychological safety.

According to interviews, team members felt more psychologically safe speaking up about certain topics. All teams prioritised patient safety and felt comfortable speaking up about concerns related to patient safety, indicating a sense of psychological safety [19]. However, silence was observed in teams A, B and C, there were lower survey scores for questions referring to speaking up about personal issues or disagreements, and interviews indicated that team members found speaking up about conflict or personal issues difficult. While junior team members could ask senior members for help and advice, they found it difficult to raise issues that could be deemed as challenging or confrontational. The influence of hierarchy was seen in all teams. This corresponds with research illustrating that those with higher status reported higher levels of psychological safety [26, 27, 38]. While team members found it difficult to engage with conflict or disagreements during team meetings, some found it easier during one-to-one discussions with either the team leader or another team member involved in the issue. Learning behaviours were displayed throughout team observations, and team members’ survey responses indicated that they could ask questions and share ideas. Interviews highlight that teams B, C and D are going through transitions, engaging in more learning initiatives, and becoming more psychologically safe. The leaders of teams B and C are both actively creating an open and inclusive team environment. According to interviews, the leader of team C engages in inclusive leadership by explicitly asking team members for their input and sharing her mistakes with the team in order to role model speaking up behaviour. Research has shown that doing this encourages psychological safety [18, 39–41]. In team B, the leader introduced protected time for developing teamwork and discussing concerns or mistakes made. While interviews indicated that team D has become more open, team members highlighted the need for protected time during team meetings for people to raise issues that were important to them, to connect to one another and to discuss their experiences that week.

All leaders displayed supportive behaviour during observations. The leader of team B showed the most supportive behaviour, using inclusive language throughout the meeting. Survey and interview data indicated that team members felt supported by their team leaders. However, interviews highlighted that there were still examples of team members not feeling comfortable taking interpersonal risks. While past research has highlighted the role of supportive leaders play in promoting psychological safety [23–25], supportive leadership alone is not enough. As discussed above, there were still issues that team members didn’t feel comfortable discussing and the impact of hierarchy and historical cultures of fear could still be seen.

Interpersonal dynamics also influenced psychological safety. As outlined in previous literature, peer support improves psychological safety within teams [10]. During observations of team B, team members showed lower supportive behaviours than their team leader and, during interviews, only one team member mentioned receiving peer support. Lower levels of peer support were found in the multidisciplinary team, where team members worked in different departments. When team members worked within the same discipline, it was easier for them to support one another in their roles and this facilitated psychological safety.

Since members of team B had been working in the hospital for a long time, they were familiar with one another. However, on teams A and D, some team members worked separately from the rest of the team. As a result, they were less familiar with and comfortable around other team members. A member of team C suggested that if team members were given time to get to know one another without the presence of their leader, they would become more comfortable and issues could be discussed more openly. Having close and connected working roles facilitates familiarity and, as a result, psychological safety. This highlights the need for teams whose roles are more separate from one another to make deliberate efforts to cultivate familiarity. This corresponds with the need to develop interventions which are suited for use across and between multiple disciplines [8]. This is particularly important in a healthcare context, where psychological safety is needed for “teaming”, an active process which allows multidisciplinary healthcare teams to work together to deliver increasingly complex patient care [10, 42].

### Implications for practice and future research

In addition to gaining an understanding of psychological safety within healthcare teams, this study aimed to inform the development of interventions to improve it. A recent systematic review of such interventions has highlighted that in order to improve their effectiveness, interventions targeting psychological safety need to be grounded in the experiences of the target audience [8]. This study contributes to a more in-depth understanding of psychological safety within healthcare teams which is needed to develop future interventions. Compared to observation and survey data, the data collected through interviews with team members provided the most valuable insights into the specific areas which interventions can target. This is because participants were given the opportunity to discuss areas in which they thought psychological safety in their team could be improved. Firstly, the issue of time was raised in each team. This mainly involved giving time during meetings for discussing more personal issues or experiences rather than only focusing on operational issues. This had already been done within team B and the team leader had noticed some improvements in trust and openness as a result. Time was also needed for prioritising learning and becoming more familiar with one another. Secondly, since team members felt more psychologically safe talking about difficult subjects during one-to-one interactions, these opportunities for interactions outside of a team setting should be encouraged. Interviews also highlighted the need to build relationships and foster familiarity with new team members and team members who work separately from the rest of the team. Lastly, interventions should encourage an awareness that all team members play a valuable role and explicitly ask for input from team members who are more vulnerable to low psychological safety, such as junior team members.

For future research, it is important to note the impact different data collection methods has on our understanding of psychological safety. As can be seen from Fig. 1, surveys provided an overview of the levels of psychological safety within the teams, however, observations and interviews provide more detailed and nuanced understanding. Observations provided a more objective view of behaviours relating to psychological safety [34] and interviews offered insight into team members’ past and present experiences both within and outside team meetings. Researchers should be aware of the different levels of understanding gained from the use of these different methods of data collection and use this to ensure the type of data they collect is suited to their research question.

### Strengths and limitations

This study combined survey, observation and interview data to gain an in-depth understanding of psychological safety within four healthcare teams. The deliberate inclusion of common components across the survey, observations, and interview analysis facilitated the triangulation of data. This provided a more detailed and holistic understanding of psychological safety.

However, some limitations must also be noted. Data was collected within one case study hospital, restricting the generalisability of findings. To address this, we have presented detailed analysis and given as much contextual information as possible for each team, without compromising the anonymity of participants. This should allow readers to determine whether they are applicable in other settings [32, 43].

Through examining the different outputs using multiple methods of data collection, this study provides a more in-depth understanding of psychological safety in healthcare teams. Within observations, it was difficult to accurately observe silence, count individual episodes of silence and determine the motivation behind silence. However, interviews were a valuable source for exploring team members’ reasons for remaining silent. While both interviews and surveys are vulnerable to self-report bias [44], the observation measure offers a more objective measure of psychological safety. The observation measure also provides information on team-level dynamics related to psychological safety. This team-level understanding aided our analysis of interviews in order to capture individual team members’ perceptions of psychological safety. This understanding is important since the overarching aim of the programme of research (of which this study is one component) is to inform the design of an intervention to improve psychological safety at the team level. In order to calculate inter-rater reliability for the observation measure without inhibiting teams by having multiple coders present, future research could use a video camera to record meetings [45, 46].

## Conclusion

This study examines psychological safety within four healthcare teams. Results from surveys, observations and interviews are considered together in order to gain an in-depth understanding of psychological safety within these teams. Based on our findings, recommendations are made for future research and the development of interventions to improve psychological safety.

## Supplementary information

**Additional file 1.**

## Data Availability

The datasets used and/or analysed during the current study are available from the corresponding author on reasonable request.
