# Supplementary material for: Exploring psychological safety in healthcare teams to inform the development of interventions: combining observational, survey and interview data
Source: BMC Health Serv Res. 2020 Aug 31;20:810. doi: 10.1186/s12913-020-05646-z (PMC7456753; doi:10.1186/s12913-020-05646-z)
Supplement: Supplementary file 1 — Additional file 1. [file 12913_2020_5646_MOESM1_ESM.docx]

**Supplementary File**

| **Interview Schedule** |
| --- |
| **Questions for all participants (part 1)** |
| In general, how well do people on this team work together?  *Follow up:* What is the atmosphere like during team meetings/interactions? |
| Can you tell me about a time when you felt comfortable to be your authentic self within this team? |
| Can you tell me about a time when you felt you needed to hold back within this team? i.e. you could not share your opinion/be honest |
| Do you think members of the team are honest with each other? |
| Do difficult issues get discussed within this team? |
| Is there ever confrontation or conflict within this team? If so, how does the team deal with this? |
| **Questions for team leader only** |
| As the leader of this team, do you think team members feel comfortable sharing their opinions with you?  *Follow up:* If so, what kinds of issues do they speak up to you about? Do you think they feel comfortable disagreeing with you or challenging you? |
| Do you think there is anything that team members would feel less comfortable speaking up about /discussing during team meetings?  *Follow up:* Do team members ever approach you to talk about these issues outside of team meetings? |
| Do team members voice different opinions to you in private compared to during team meetings? |
| **Questions for team members only** |
| Do you feel comfortable speaking up/sharing your opinion with your team leader? If so, what kinds of issues do you feel comfortable speaking up about? |
| Is there anything that you would feel less comfortable speaking up to your team leader about?  *Follow up:* If not, why not? |
| Do you feel comfortable disagreeing with or challenging your team leader? Do you think other team members do? |
| Would you share different opinions with your leader in private compared to during team meetings? |
| **Questions for all participants (part 2)** |
| How do you feel about speaking up to other team members?  *Follow up:* Are there any team members who you would feel less comfortable speaking up to/sharing your opinions with? Why? In general, are there any issues you would feel less comfortable discussing in your team? Why? |
| Can you tell me about one of your experiences speaking up/sharing your opinion in this team?  *Cues: A time when you chose not to speak up/share your opinions? A time you felt completely comfortable to speak up/share your opinion?* |
| Do you always agree with decisions taken by this team?  If not, would you openly disagree with the decision? |
| If you disagree with someone's opinion in a meeting, are you more likely to say so in the meeting or discuss your concerns with one or two people after the meeting? |
| Do you think everyone’s opinion is valued in this team?  *Follow up:* If so/if not, how is this communicated to all team members? |
| What do you think could make team members more comfortable to be their authentic selves/speak up/share their opinions in this team? |
